# Logic of approximate entailment in quasimetric and in metric spaces

**DOI:** 10.1007/s00500-016-2215-x

**Published:** 2016-06-17

**Authors:** Thomas Vetterlein

**Affiliations:** Department of Knowledge-Based Mathematical Systems, Johannes Kepler University Linz, Altenberger Straße 69, 4040 Linz, Austria

## Abstract

It is known that a quasimetric space can be represented by means of a metric space; the points of the former space become closed subsets of the latter one, and the role of the quasimetric is assumed by the Hausdorff quasidistance. In this paper, we show that, in a slightly more special context, a sharpened version of this representation theorem holds. Namely, we assume a quasimetric to fulfil separability in the original sense due to Wilson. Then any quasimetric space can be represented by means of a metric space such that distinct points are assigned disjoint closed subsets.

This result is tailored to the solution of an open problem from the area of approximate reasoning. Following the lines of E. Ruspini’s work, the Logic of Approximate Entailment ($\mathsf {LAE}$) is based on a graded version of the classical entailment relation. We present a proof calculus for $\mathsf {LAE}$ and show its completeness with regard to finite theories.

## Introduction

A quasimetric is defined similarly to a metric; the assumption of symmetry, however, is dropped. This generalisation of the concept of a distance naturally occurs in many real-world situations. An often cited example is the time that a walker needs to get from one place to another one within a mountainous area. Quasimetric spaces are moreover closely related to weighted directed graphs. The latter play a significant role in computer science, in particular for the formulation of network flow problems (Ahuja et al. 1993; Heineman et al. 2009).

It is certainly also true that the notion of a quasimetric is by far less common in mathematics than its symmetric counterpart. Remarkably, however, the metric spaces themselves give rise to a non-symmetric distance function. The *Hausdorff quasidistance* quantifies the difference between subsets of a metric space, rather than between its points. It shares with a metric certain characteristic properties like the triangle inequality, but symmetry does in general not hold. Hyperspaces consisting of subsets of a metric space, endowed with the Hausdorff quasidistance, are used in a number of contexts. An example is the area of point-free geometry (Concilio and Gerla 2006; Concilio 2013). Measuring the degree of distinctness between subsets of a metric space is moreover an issue in fuzzy set theory; see, for example, Gerla (2004).

It is reasonable to ask whether distance functions that violate symmetry but otherwise resemble a metric always arise from a metric space in the above way mentioned. We find the affirmative answer in Vitolo’s paper (1995). Vitolo has studied spaces (*W*, *q*), where $q :W \times W \rightarrow {\mathbb R}^+$ fulfils the triangle inequality as well as the following version of the separation axiom: $q(a,b) = q(b,a) = 0$ if and only if $a = b$. He proved that any such space can be embedded into the hyperspace of non-empty closed sets of a metric space, the role of the quasimetric being taken by the Hausdorff quasidistance. Also among the results in Gerla (2004), Vitolo’s representation theorem can be found.

In the present paper, we study distance functions of a more special type. In this paper, a quasimetric is understood to be a mapping $q :W \times W \rightarrow \bar{\mathbb R}^+$ such that, for $a, b, c \in W$, $q(a,c) \leqslant q(a,b) + q(b,c)$, and $q(a,b) = 0$ if and only if $a = b$. This definition is in accordance with the early work of Wilson on the topic (1931); however, it is evidently stronger than the one used in Vitolo (1995) and Gerla (2004). We prove a representation theorem similar to Vitolo’s, but with certain additional features.

The motivation underlying Vitolo’s work was to characterise the quasimetrisability of a topological space. Gerla’s interest originated from the connection between quasimetrics and fuzzy orders. The results of the present paper are developed yet for another purpose: we are interested in logics for approximate reasoning, in line with the framework proposed in Ruspini’s seminal paper (1991).

Recall that in classical propositional logic, properties are modelled by subsets and the logical entailment corresponds to the subsethood relation. Following Ruspini (1991), a graded version of the subsethood relation may be used in order to express the approximate entailment of properties. To this end, we endow the underlying space with a similarity function. A similarity function is a flexible tool that allows the quantification of the distinctness of points. One possibility is to take (the dual of) a metric, and we presuppose this choice here. Then the Hausdorff quasidistance associated with the metric provides a means of expressing that one subset is contained in another one to a certain degree.

To capture this idea on the basis of a logical calculus turns out to be difficult. We focus here on the so-called Logic of Approximate Entailment, $\mathsf {LAE}$ for short, which was introduced by Rodríguez in his PhD thesis (2002). The completeness of $\mathsf {LAE}$ in a finitary setting has long been known; see Rodríguez (2002), cf. also Esteva et al. (2012). But an axiomatisation of $\mathsf {LAE}$ in a more general setting has remained an open problem; cf. Rodríguez (2002, Section 8.3).

In our previous paper Vetterlein (2015), we have dealt with a closely related setting. Namely, we have considered a modification of $\mathsf {LAE}$, the logic $\mathsf {LAE}^q$, which is based on quasimetric spaces instead of metric spaces. We have shown that a certain calculus, denoted by $\mathbf {LAE}$ and consisting of only six rules (Vetterlein 2015), is sound and complete for $\mathsf {LAE}^q$. The question has been open if this result could be useful also for the logic $\mathsf {LAE}$. To this end, a means of representing a quasimetric space within a metric space is required. Indeed, what we need is a representation theorem similar to the one given in Vitolo (1995). Vitolo’s result itself is, unfortunately, not applicable. The crucial problem is that points are assigned subsets of a metric space and subsets assigned to distinct points are in general not disjoint. The present paper offers a solution for this situation.

Our core result is the following. For any quasimetric space (*X*, *q*), there is a metric space (*Y*, *d*) and a mapping $\iota :X \rightarrow {{\mathcal P}}(Y)$ such that the following holds: $\iota$ maps distinct points to disjoint closed subsets of *Y* and under this embedding the quasimetric on *X* coincides with the Hausdorff quasidistance on *Y*. We in fact even show that the natural extension of $\iota$ to the power set of *X* preserves the Hausdorff quasidistance. We apply this result in order to show that the calculus $\mathbf {LAE}$, which is known to be complete for the logic $\mathsf {LAE}^q$ based on quasimetric spaces, is actually complete for $\mathsf {LAE}$, which is based on metric spaces. We may consequently say that we are able to define a logical calculus that is well in line with the framework for approximate reasoning in its original form due to Ruspini.

The paper is structured as follows. In Sect. 2, we introduce the main notions under consideration. Section 3 contains the representation theorem for quasimetric spaces. We apply this result in Sect. 4 to the Logic of Approximate Entailment $\mathsf {LAE}$, showing that the calculus $\mathbf {LAE}$ from Vetterlein (2015) is sound and complete for $\mathsf {LAE}$. An outlook on possible further work is contained in the concluding Sect. 5.

## Quasimetric spaces and metric spaces

We start by fixing notation and terminology used in this paper.

We let ${\mathbb R}^+ = \{ r \in {\mathbb R}:r \geqslant 0 \}$ be the set of positive reals, and we denote by $\bar{\mathbb R}^+$ the extended set of positive reals, that is, $\bar{\mathbb R}^+= {\mathbb R}^+ \cup \{\infty \}$. Here, $\infty$ is a new element such that $r < \infty$ for any $r \in {\mathbb R}^+$, and we define $r + \infty = \infty + r = \infty$ for any $r \in \bar{\mathbb R}^+$.

### Definition 2.1

Let *X* be a non-empty set. A *quasimetric* on *X* is a mapping $q :X \times X \rightarrow \bar{\mathbb R}^+$ such that the following conditions hold:

(M1): For any $a \in X$, we have $q(a,a) = 0$.
(M2): For any $a, b \in X$, $\;q(a,b) = 0$ implies $a = b$.
(M3): For any $a, b, c \in X$, we have $q(a,c) \leqslant q(a,b) + q(b,c)$.

In this case, we refer to (*X*, *q*) as a *quasimetric space*.

Moreover, we call *q* a *metric* and (*X*, *q*) a *metric space* if, in addition, the following condition holds:

(M4): For any $a, b \in X$, we have $q(a,b) = q(b,a)$.

As mentioned in the introduction, there is a close relationship between quasimetric spaces on the one hand and weighted directed graphs on the other hand. The details are as follows.

### Remark 2.2

Consider a finite directed graph, consisting of the vertices *V* and edges $E \subseteq V \times V$, together with a weight function $w :E \rightarrow {\mathbb R}^+$ such that, for any $(v_1,v_2) \in E$, $w(v_1,v_2) = 0$ if and only if $v_1 = v_2$. Define the length of a directed path as the sum of the weights of its edges. Then, for any pair of vertices $v_1, v_2 \in V$, let $q(v_1,v_2) = 0$ if $v_1 = v_2$, let $q(v_1,v_2)$ be the minimal length of a path from $v_1$ to $v_2$ if $v_1 \ne v_2$ and there is at least one path from $v_1$ to $v_2$, and let $q(v_1,v_2) = \infty$ if $v_1 \ne v_2$ and there is no path from $v_1$ to $v_2$. We easily verify that *q* makes *V* into a quasimetric space.

Assuming that the length of the paths between each two distinct vertices is lower bounded by a strictly positive value, we may generalise the observation to the infinite case.

Conversely, we may associate with each quasimetric space (*X*, *q*) the directed graph whose set of vertices is *X* and whose set of edges is $\{ (v_1,v_2) \in X \times X :q(v_1,v_2) < \infty \}$. From this graph, together with the weight function based on *q*, (*X*, *q*) arises as indicated above.

In contrast to the case of a metric, the definition of a quasimetric is in the literature not uniform. Most remarkably, instead of the separability axiom (M2), the following one is often used:

(M2$$^\prime $$)
For any $a, b \in X$, $\;q(a,b) = q(b,a) = 0$ implies $a = b$.

Assuming (M2$^\prime$), we evidently could not conclude from $q(a,b) = 0$ or $q(b,a) = 0$ alone that *a* and *b* coincide. Wilson (1931), who is reportedly the first to study quasimetric spaces on an axiomatic basis, uses condition (M2) as well. In Vitolo (1995) and Gerla (2004), however, (M2$^\prime$) is used.

A further difference is that a quasimetric is often defined to take values in ${\mathbb R}^+$ only. Although this deviation might sound less significant, we should note that the procedure presented in the subsequent section relies on it in an essential way.

We endow a quasimetric space (*X*, *q*) with a topology in the usual way: its basis consists of the sets of the form $\{ b \in X :q(a, b) < \varepsilon \}$, where $a \in X$ and $\varepsilon > 0$. By (M2), *X* is a $T_1$-space; hence, all singletons are closed. Note that (M2$^\prime$) would imply not more than $T_0$.

### Definition 2.3

Let (*X*, *q*) be a quasimetric space. For a point $a \in X$ and a set $B \subseteq X$, we put

$$\begin{aligned} p_q(a,B) \;=\; \inf _{b \in B} \; q(a,b), \end{aligned}$$

and we define the *Hausdorff quasidistance* between subsets *A* and *B* of *X* by

$$\begin{aligned} e_q(A,B) \;=\; \sup _{a \in A} \; p_q(a,B). \end{aligned}$$

We compile a few straightforward facts on the Hausdorff quasidistance.

### Lemma 2.4

Let (*X*, *q*) be a quasimetric space. Then we have, for any $A, A_\iota , B, C \subseteq X$, $\iota \in I$:

(i) $e_q(A,B) = 0$ if $A \subseteq B$. If *B* is closed, also the converse holds.
(ii) $$e_q(\bigcup _\iota A_\iota , B) \;=\; \sup _\iota \; e_q(A_\iota , B)$$.
(iii) $$e_q(A, B) \geqslant e_q(A, C)$$ if $$B \subseteq C$$.
(iv) $$e_q(A,C) \leqslant e_q(A,B) + e_q(B,C)$$.
(v) $e_q(\varnothing , B) = 0$. Moreover, if *A* is non-empty, $e_q(A, \varnothing )$ $= \infty$.

### Proof

The first part of (i) and (ii), (iii), (v) are clear.

For the second part of (i), assume that $e_q(A,B) = 0$ and *B* is closed. Then $p_q(a,B) = 0$ for any $a \in A$ and since *B* is closed, it follows that $a \in B$ for any $a \in A$, that is, $A \subseteq B$.

To verify (iv), assume that *A*, *B*, *C* are non-empty; otherwise, the inequality is clear from (v). We calculate

$$\begin{aligned} e_q(A, B) + e_q(B, C)= & {} \sup _{a \in A} \inf _{b \in B} q(a,b) + \sup _{b \in B} \inf _{c \in C} q(b,c) \\= & {} \sup _{a \in A} \inf _{b \in B} (q(a,b) + \sup _{b' \in B} \inf _{c \in C} q(b',c)) \\\geqslant & {} \sup _{a \in A} \inf _{b \in B} (q(a,b) + \inf _{c \in C} q(b,c)) \\= & {} \sup _{a \in A} \inf _{b \in B} \inf _{c \in C} (q(a,b) + q(b,c)) \\\geqslant & {} \sup _{a \in A} \inf _{c \in C} q(a,c) \;=\; e_q(A, C), \end{aligned}$$

using (M3). $$\square $$

By Lemma 2.4, the Hausdorff quasidistance $e_q$, defined on the hyperspace of closed sets of a quasimetric space *X*, fulfils conditions (M1), (M2$^\prime$), and (M3). This is actually the guiding example in Vitolo (1995). From Lemma 2.4(i), however, it is also clear that condition (M2) is in general violated.

The situation is different for hyperspaces consisting of mutually disjoint subsets of a metric space. Our own guiding example of a quasimetric space is the following one, which is given in Wilson (1931) right at the beginning.

### Lemma 2.5

Let (*X*, *d*) be a metric space, let ${{\mathcal Y}}$ be a partition of *X* into non-empty closed subsets, and let $e_d$ be the Hausdorff quasidistance restricted to ${{\mathcal Y}}$. Then $({{\mathcal Y}}, e_d)$ is a quasimetric space.

### Proof

Let $A, B \in {{\mathcal Y}}$. Clearly, $e_d(A,A) = 0$, that is, (M1) holds. Furthermore, $e_d(A,B) = 0$ implies $A \subseteq B$ by Lemma 2.4(i). By assumption, *A* and *B* are non-empty subsets of *X* that are either equal or disjoint. Hence, $A = B$ and (M2) follows. (M3) holds by Lemma 2.4(iv). $\square$

We will see that any quasimetric space arises in this way from a metric.

## Representation of quasimetric spaces

In this section, we show how quasimetric spaces can be led back to metric spaces. Intuitively speaking, we “expand” each point of a quasimetric space to a set of possibly infinite cardinality and we endow the (disjoint) union of all these sets with a metric. This is done such that the quasimetric between two points of the original space becomes the Hausdorff quasidistance between the two associated subsets of the new space. We shall furthermore see that our representation even preserves the Hausdorff quasidistance on the original space.

We start with an auxiliary lemma. By a *pseudometric*, we mean a mapping $q :X \times X \rightarrow \bar{\mathbb R}^+$ fulfilling (M1), (M3), and (M4).

### Lemma 3.1

Let *X* be a non-empty set and let $D \subseteq X \times X$ be such that $(a,a) \in D$ for all $a \in X$, and $(a,b) \in D$ implies $(b,a) \in D$ for any $a, b \in X$. Let ${\hat{d}} :D \rightarrow \bar{\mathbb R}^+$ be such that the following conditions hold:

($$\alpha $$)
Let $a, b \in X$. Then $(a,b) \in D$ and ${\hat{d}}(a,b) = 0$ if and only if $a = b$.
($$\beta $$)
Let $$(a,b) \in D$$. Then $${\hat{d}}(a,b) = {\hat{d}}(b,a)$$.
($$\gamma $$)
Let $a_0, \ldots , a_n \in X$ be pairwise distinct. If $(a_0, a_1), \ldots ,$ $(a_{n-1}, a_n)$, where $n \geqslant 2$, as well as $(a_0, a_n)$ are in *D*,  then ${\hat{d}}(a_0, a_n) \leqslant {\hat{d}}(a_0, a_1) + \cdots + {\hat{d}}(a_{n-1}, a_n)$.

Moreover, let $d :X \times X \rightarrow \bar{\mathbb R}^+$ be defined as follows. For $a, b \in X$, let $d(a,b) = 0$ if $a = b$ and otherwise

1 $$\begin{aligned} d(a, b) =&\inf \{ {{\hat{d}}}(c_0,c_1) + \cdots + {\hat{d}}(c_{n-1},c_n) :\nonumber \\&\quad \text {there are pairwise distinct elements } c_0, \ldots , c_n \nonumber \\&\quad (n \geqslant 1) \hbox { in } X \hbox {such that} a=c_0, \;b = c_n, \text { and } \nonumber \\&\quad (c_0, c_1), \ldots , (c_{n-1},c_n) \in D \}. \end{aligned}$$

Then *d* is the largest pseudometric on *X* extending ${\hat{d}}$.

### Proof

We claim that $d' :X \times X \rightarrow \bar{\mathbb R}^+$ defined by

$$\begin{aligned} d'(a, b) =&\inf \{ {{\hat{d}}}(c_0,c_1) + \cdots + {{\hat{d}}}(c_{n-1},c_n) :\\&\quad c_0, \ldots , c_n \in X \hbox { such that } a = c_0, \;b = c_n, \\&\quad \hbox {and } (c_0, c_1), \ldots , (c_{n-1}, c_n) \in D \} \end{aligned}$$

coincides with *d*. In fact, let $a, b \in X$. If $a = b$, we have $d'(a,b) = 0 = d(a,b)$ by ($\alpha$). Otherwise, consider a sequence $a = c_0, \ldots , c_n = b$ such that all pairs of successive elements are in *D*. Then we can determine a subsequence as follows: if $c_i = c_j$ for some $0 \leqslant i < j \leqslant n$, we remove the elements $c_{i+1}, \ldots , c_j$, and we repeat doing so until no element appears twice. Let $c'_0, \ldots , c'_m$ be the resulting sequence. Then the first and last elements are the same as before, that is, $c'_0 = c_0 = a$ and $c'_m = c_n = b$. Furthermore, pairs of successive elements are still in *D*. As ${\hat{d}}(c'_0,c'_1) + \cdots + {\hat{d}}(c'_{m-1},c'_m) \leqslant {\hat{d}}(c_0,c_1) + \cdots + {\hat{d}}(c_{n-1},c_n)$, we conclude $d = d'$.

It is readily checked that $d'$ and hence *d* is a pseudometric. Furthermore, by ($\gamma$), *d* extends ${\hat{d}}$. By construction, *d* is the largest pseudometric extending ${\hat{d}}$. $\square$

We now turn to the core result of the present paper. We will denote by $A^{<\omega }$ the set of all finite sequences of elements of a set *A*. A finite sequence $(a_1, \ldots , a_n)$ is said to *extend* another one $(b_1, \ldots , b_m)$ if $m \leqslant n$ and $a_1 = b_1, \ldots , b_m = a_m$. Furthermore, we write $\varnothing$ for the sequence of length 0, and for $\sigma \in A^{<\omega }$ and $a \in A$, we denote by $\sigma \mathbin {\!^\smallfrown \!}a$ the sequence arising from $\sigma$ by adding *a* as the new last element.

### Theorem 3.2

Let (*X*, *q*) be a quasimetric space. Then there is a metric space (*Y*, *d*) and a mapping $\iota :X \rightarrow {{\mathcal P}}(Y)$ such that $\{ \iota (a) :a \in X \}$ is a partition of *Y* and

2 $$\begin{aligned} q(a,b) \;=\; e_d(\iota (a), \iota (b)) \end{aligned}$$

holds for any $a, b \in X$.

### Proof

The assertion is trivial if *q* is actually a metric. Assume that this is not the case.

Let

$$\begin{aligned} U \;=\; \{ (a,b) \in X \times X :q(a,b) < q(b,a) \} \end{aligned}$$

and put

$$\begin{aligned} Y \;=\; X \times U^{<\omega }. \end{aligned}$$

Let *D* be the subset of $Y \times Y$ consisting of all pairs of the following form:

$$\begin{aligned}&((a,\sigma ), (b,\sigma )), \;\text {where } a, b \in X \hbox { and } \sigma \in U^{<\omega }, \\ \text {or}\;\;&((a, \sigma ), (b, \sigma \mathbin {\!^\smallfrown \!}(a,b))), \;\text {where } \sigma \in U^{<\omega } \hbox { and } (a, b) \in U, \\ \text {or}\;\;&((b, \sigma \mathbin {\!^\smallfrown \!}(a,b)), (a,\sigma )), \;\text {where } \sigma \in U^{<\omega } \hbox { and } (a, b) \in U. \end{aligned}$$

Let us define a map ${\hat{d}} :D \rightarrow \bar{\mathbb R}^+$ as follows. For $a, b \in X$ and $\sigma \in U^{<\omega }$, let

$$\begin{aligned} {\hat{d}}((a,\sigma ), (b,\sigma )) \;=\; \max \,\{ q(a,b), \, q(b,a) \}; \end{aligned}$$

and for $\sigma \in U^{<\omega }$ and $(a,b) \in U$, let

$$\begin{aligned} {\hat{d}}((a, \sigma ), (b, \sigma \mathbin {\!^\smallfrown \!}(a,b)))&\;=\; {\hat{d}}((b, \sigma \mathbin {\!^\smallfrown \!}(a,b)), (a,\sigma )) \nonumber \\&\;=\; q(a,b). \end{aligned}$$

Our aim is to show that Lemma 3.1 is applicable to ${\hat{d}}$. It is clear that the domain $D \subseteq Y \times Y$ of ${\hat{d}}$ fulfils the requirements. Moreover, we easily check that ${\hat{d}}$ fulfils conditions ($\alpha$) and ($\beta$).

Before turning to condition ($\gamma$), we show two auxiliary facts.

(A) Let $(x_1, \sigma _1), \ldots , (x_n, \sigma _n)$, where $n \geqslant 2$, be a finite sequence of pairwise distinct elements of *Y*. Assume that ${\hat{d}}$ is defined for each pair of successive elements and that $\sigma _n$ extends $\sigma _1$. Then, for each $i = 1, \ldots , n-1$, $\sigma _{i+1}$ extends $\sigma _i$. Indeed, assume the contrary. We have that, for each $i = 1, \ldots , n-1$, either $\sigma _i = \sigma _{i+1}$, or $\sigma _{i+1}$ extends $\sigma _i$ by one element, or $\sigma _i$ extends $\sigma _{i+1}$ by one element. Since $\sigma _n$ extends $\sigma _1$, we conclude that the sequence contains two pairs of successive elements such that one is of the form $(x, \sigma \mathbin {\!^\smallfrown \!}(a,b)), (y, \sigma )$ and the other one $(x', \sigma ), (y', \sigma \mathbin {\!^\smallfrown \!}(a,b))$. Since these two pairs are in *D*, we conclude $x = y' = b$ and $y = x' = a$, in contradiction to our assumption that the elements of the sequence are pairwise distinct.
(B) Let $(x_0, \sigma _0), \ldots , (x_n, \sigma _n)$, where $n \geqslant 1$, be a finite sequence of pairwise distinct elements of *Y*. Assume that ${\hat{d}}$ is defined for each pair of successive elements and that $\sigma _n$ extends $\sigma _0$. Then

$$\begin{aligned}&q(x_0,x_n) \leqslant {\hat{d}}((x_0, \sigma _0), (x_1, \sigma _1)) + \cdots \\&\quad +\,{\hat{d}}((x_{n-1}, \sigma _{n-1}), (x_n, \sigma _n)). \end{aligned}$$

Indeed, by (A), $\sigma _{i+1}$ extends $\sigma _i$ for each $i = 0, \ldots , n-1$. By the definition of ${\hat{d}}$, it follows that $q(x_i, x_{i+1}) \leqslant {\hat{d}}((x_i, \sigma _i), (x_{i+1}, \sigma _{i+1}))$ for each $i = 0, \ldots , n-1$. Hence,

$$\begin{aligned} q(x_0, x_n) \;\leqslant \;&q(x_0, x_1) + \cdots + q(x_{n-1}, x_n) \\ \leqslant \;&{\hat{d}}((x_0, \sigma _0), (x_1, \sigma _1)) + \cdots \\&+\,{\hat{d}}((x_{n-1}, \sigma _{n-1}), (x_n, \sigma _n)), \end{aligned}$$

as asserted.

To see ($\gamma$), we have to show

3 $$\begin{aligned}&{\hat{d}}((x_0, \sigma _0), (x_n, \sigma _n)) \leqslant {\hat{d}}((x_0, \sigma _0), (x_1, \sigma _1)) + \cdots \nonumber \\&\quad +\, {\hat{d}}((x_{n-1}, \sigma _{n-1}), (x_n, \sigma _n)), \end{aligned}$$

provided that $(x_0, \sigma _0), \ldots , (x_n, \sigma _n)$, where $n \geqslant 2$, are pairwise distinct and that ${\hat{d}}$ is defined in each indicated case.

We have that either $\sigma _n$ extends $\sigma _0$ or vice versa. Converting the order of the sequence if necessary, we may assume that the former possibility applies, that is, $\sigma _n$ extends $\sigma _0$. Consequently, by (B), we have

4 $$\begin{aligned}&{}q(x_0, x_n) \leqslant {\hat{d}}((x_0, \sigma _0), (x_1, \sigma _1)) +\cdots \nonumber \\&\quad +\, {\hat{d}}((x_{n-1}, \sigma _{n-1}), (x_n, \sigma _n)). \end{aligned}$$

To see (3), we distinguish two cases.

**Case 1** $\sigma _0$ and $\sigma _n$ coincide. By (A), we then have $\sigma _0 = \sigma _1 = \cdots = \sigma _n$. Moreover, by (B), (4) holds with “$q(x_0, x_n)$” being replaced by “$q(x_n, x_0)$”. As ${\hat{d}}((x_0, \sigma _0), (x_n, \sigma _n)) = \max \, \{ q(x_0, x_n), \, q(x_n, x_0) \}$, we conclude (3).

**Case 2** $\sigma _n$ extends $\sigma _0$ by one element. Then ${\hat{d}}((x_0, \sigma _0),$ $(x_n, \sigma _n)) = q(x_0, x_n)$ and (3) holds again by (4).

The proof is complete that ${\hat{d}}$ fulfils property ($\gamma$) of Lemma 3.1. We conclude that there is a largest pseudometric *d* extending ${\hat{d}}$ to the whole $Y \times Y$, given by (1).

We claim that *d* is in fact a metric. Let $(a,\sigma ), (b,\sigma ') \in Y$ be distinct. We have to show $d((a,\sigma ), (b,\sigma ')) > 0$. If $\sigma = \sigma '$, the pair is in *D* and the assertion is clear by (M2). Assume that $\sigma$ and $\sigma '$ are distinct. W.l.o.g., we can then assume that there is a $(c,d) \in U$ that occurs in $\sigma '$ but not in $\sigma$. Let $(a, \sigma ) = (x_0, \sigma _0), \ldots , (x_n, \sigma _n) = (b, \sigma ')$ be such that ${\hat{d}}$ is defined for each pair of successive elements. Then there is a $0 \leqslant i \leqslant n-1$ such that $\sigma _{i+1} = \sigma _i \mathbin {\!^\smallfrown \!}(c,d)$ and this means ${\hat{d}}((x_i, \sigma _i), (x_{i+1}, \sigma _{i+1})) = q(c,d)$. It follows that $d((a, \sigma ), (b,\sigma ') \geqslant q(c,d)$ and since $c \ne d$ the assertion follows from (M2).

We next note:

(C) For any $(a, \sigma ), (b, \sigma ') \in Y$ such that $\sigma '$ extends $\sigma$, we have
  $$\begin{aligned} q(a,b) \leqslant d((a, \sigma ), (b, \sigma ')). \end{aligned}$$

Indeed, this is immediate from (B).

We now define

$$\begin{aligned} \iota :X \rightarrow {{\mathcal P}}(Y) ,a \mapsto \{ (a, \sigma ) :\sigma \in U^{<\omega } \}. \end{aligned}$$

Let $a, b \in X$; we have to show (2). By (C), we have for any $\sigma \in U^{<\omega }$

$$\begin{aligned} d((a, \varnothing ), (b, \sigma )) \geqslant q(a,b), \end{aligned}$$

and it follows that $e_d(\iota (a), \iota (b)) \geqslant q(a,b)$. In order to prove the converse inequality, let us first assume that $q(a,b) < q(b,a)$. Then $(a,b) \in U$ and, for any $\sigma \in U^{<\omega }$,

$$\begin{aligned} d((a, \sigma ), (b, \sigma \mathbin {\!^\smallfrown \!}(a,b))) \;=\; q(a,b), \end{aligned}$$

hence $e_d(\iota (a), \iota (b)) \leqslant q(a,b)$. Assume second that $q(a,b) \geqslant q(b,a)$. Since, for any $\sigma \in U^{<\omega }$,

$$\begin{aligned} d((a, \sigma ), (b, \sigma )) \;=\; \max \, \{ q(a,b), \, q(b,a) \} \;=\; q(a,b), \end{aligned}$$

we again have $e_d(\iota (a), \iota (b)) \leqslant q(a,b)$. The proof of (2) is complete. $\square$

The remainder of this section is devoted to several refinements of Theorem 3.2. We could have integrated the following lemmas directly into Theorem 3.2; we did not do so in order to avoid the lengthy proof to be even further extended.

We first consider the topologies on the represented and representing spaces.

### Lemma 3.3

Let (*X*, *q*) be a quasimetric space. Let $\iota :X \rightarrow {{\mathcal P}}(Y)$ be the representation of (*X*, *q*) by means of the metric space (*Y*, *d*) according to Theorem 3.2. Then, for each $a \in X$, $\iota (a)$ is a closed subset of *Y*.

### Proof

With reference to the proof of Theorem 3.2, let $a \in X$ and let $(b,\sigma ) \in Y$ such that $a \ne b$. To show that $\iota (a) = \{ (a,\sigma ') :\sigma ' \in U^{<\omega } \}$ is closed, we will determine an $\varepsilon > 0$ such that $d((a, \sigma '), (b, \sigma )) > \varepsilon$ for any $\sigma ' \in U^{<\omega }$.

For each $\sigma ' \in U^{<\omega }$, one of the following alternatives applies:

**Case 1** $\sigma '$ extends $\sigma$. Then $d((a, \sigma '), (b, \sigma )) \geqslant q(b,a) > 0$ by (C).

**Case 2**  There is a first element $(c,d) \in U$ in $\sigma$ where $\sigma$ differs from $\sigma '$. Let then $(a, \sigma ') = (x_0, \sigma _0), \ldots , (x_n, \sigma _n) = (b, \sigma )$ be such that ${\hat{d}}$ is defined for each pair of successive elements. Then there is a $0 \leqslant i \leqslant n-1$ such that $\sigma _{i+1} = \sigma _i \mathbin {\!^\smallfrown \!}(c,d)$ and this means ${\hat{d}}((x_i, \sigma _i), (x_{i+1}, \sigma _{i+1})) = q(c,d)$. It follows that $d((a, \sigma '), (b,\sigma )) \geqslant q(c,d) > 0$.

We conclude that $d((a, \sigma '), (b, \sigma ))$ is bounded from below by finitely many nonzero values, and the assertion follows. $\square$

We conclude that any quasimetric space is of the form indicated in Lemma 2.5.

For the following lemma, recall that a topology is called discrete if any singleton and consequently any subset is open.

### Lemma 3.4

Let (*X*, *q*) be a quasimetric space. Let $\iota :X \rightarrow {{\mathcal P}}(Y)$ be the representation of (*X*, *q*) by means of the metric space (*Y*, *d*) according to Theorem 3.2. If the topology of *X* is discrete, then so is the topology of *Y*.

### Proof

We refer again to the proof of Theorem 3.2. Assume that *X* has the discrete topology; then, $\{a\}$ is open and hence there is an $\varepsilon > 0$ such that $q(a,b) \geqslant \varepsilon$ for any $b \ne a$. Let $\sigma \in U^{<\omega }$. We have to show that $\{ (a,\sigma ) \}$ is open in *Y* as well.

If $\sigma$ is of length $\geqslant 1$, let $(c,d) \in U$ be the last element of $\sigma$ and choose $0< \delta < \varepsilon \wedge q(c,d)$. If $\sigma = \varnothing$, choose $0< \delta < \varepsilon$. We claim that the $\delta$-neighbourhood of $(a, \sigma )$ is a singleton.

Let $(b, \sigma ') \in Y$ be distinct from $(a, \sigma )$ and such that ${\hat{d}}((a,\sigma ), (b,\sigma '))$ is defined. There are the following alternatives:

**Case 1** $\sigma = \sigma '$. Then $a \ne b$ and hence ${\hat{d}}((a,\sigma ), (b,\sigma ')) = \max \, \{ q(a,b), \, q(b,a) \} \geqslant q(a,b) \geqslant \varepsilon > \delta$.

**Case 2** $$\sigma ' = \sigma \mathbin {\!^\smallfrown \!}(a,b)$$. Then $${\hat{d}}((a,\sigma ), (b,\sigma ')) = q(a,b) \geqslant \varepsilon > \delta $$.

**Case 3** $\sigma = \sigma ' \mathbin {\!^\smallfrown \!}(c,d)$. Note that then $(c,d) = (b,a)$. We have ${\hat{d}}((a,\sigma ), (b,\sigma ')) = q(b,a) = q(c,d) > \delta$.

The assertion follows. $\square$

We finally show that the Hausdorff quasidistance between subsets of the represented quasimetric space is preserved.

### Lemma 3.5

Let (*X*, *q*) be a quasimetric space. Let $\iota :X \rightarrow {{\mathcal P}}(Y)$ be the representation of (*X*, *q*) by means of the metric space (*Y*, *d*) according to Theorem 3.2. Let us denote the extension of $\iota$ to subsets of *X* again by $\iota$. Then

5 $$\begin{aligned} e_q(A, B) \;=\; e_d(\iota (A), \iota (B)) \end{aligned}$$

holds for any $A, B \subseteq X$.

### Proof

The assertion is clear from Lemma 2.4(v) if *A* or *B* is empty. Let us assume that *A* and *B* are non-empty.

Let $a \in X$ and $B \subseteq X$. We will show

6 $$\begin{aligned} p_q(a, B) \;=\; e_d(\iota (a), \iota (B)); \end{aligned}$$

then (5) will follow by Lemma 2.4(ii).

By definition,

$$\begin{aligned} p_q(a, B) \;=\; \inf _{b \in B} q(a, b) \end{aligned}$$

and

$$\begin{aligned} e_d(\iota (a), \iota (B)) \;=\; \sup _{x \in \iota (a)} \inf _{y \in \iota (B)} d(x, y). \end{aligned}$$

To see that these two values coincide, we make use of the particular definition of the metric *d* from the proof of Theorem 3.2. Let $a, b \in X$ and $\sigma \in U^{<\omega }$. If $q(a,b) < q(b,a)$, we have $(a,b) \in U$ and $d((a, \sigma ), (b, \sigma \mathbin {\!^\smallfrown \!}(a,b))) = q(a,b)$. If $q(b,a) \leqslant q(a,b)$, we have $d((a, \sigma ), (b, \sigma )) = \max \, \{ q(a,b), \, q(b,a) \} = q(a,b)$. We conclude $\inf _{y \in \iota (b)}$ $d(x, y) \leqslant q(a,b)$ for any $x \in \iota (a)$ and hence

$$\begin{aligned} \inf _{y \in \iota (B)} d(x, y)&= \inf _{b \in B} \inf _{y \in \iota (b)} d(x, y) \leqslant \inf _{b \in B} q(a,b) \nonumber \\&= p_q(a, B). \end{aligned}$$

We conclude $$e_d(\iota (a), \iota (B)) \leqslant p_q(a, B)$$.

To see the converse inequality, let again $a, b \in X$. We have $d((a, \varnothing ), (b, \sigma )) \geqslant q(a,b)$ for any $\sigma \in U^{<\omega }$. Moreover, if $q(a,b) < q(b,a)$, then $(a,b) \in U$, and $d((a, \varnothing ), (b, (a,b))) = q(a,b)$. If $q(b,a) \leqslant q(a,b)$, then $d((a, \varnothing ), (b, \varnothing )) = \max \, \{ q(a,b), \, q(b,a) \} = q(a,b)$. We conclude that $\inf _{y \in \iota (b)} d((a, \varnothing ), y) = q(a,b)$. Hence

$$\begin{aligned} e_d(\iota (a), \iota (B))&\geqslant \inf _{y \in \iota (B)} d((a, \varnothing ), y) \\&= \inf _{b \in B} \inf _{y \in \iota (b)} d((a, \varnothing ), y)\\&= \inf _{b \in B} q(a,b) = p_q(a, B), \end{aligned}$$

and (6) is shown. $\square$

Our results are compiled in the following theorem.

### Theorem 3.6

Let (*X*, *q*) be a quasimetric space. Then there is a metric space (*Y*, *d*) and a mapping $\iota :X \rightarrow {{\mathcal P}}(Y)$ such that $\{ \iota (a) :a \in X \}$ is a partition of *Y* into closed subsets and $e_q(A, B) = e_d(\iota (A), \iota (B))$ holds for any $A, B \subseteq X$.

## The logic of approximate entailment

We now turn to a context in which Theorem 3.6 has an immediate consequence: to logics that allow reasoning in an approximate manner.

Following the lines of Ruspini (1991), we deal with the implication between properties up to a certain degree of tolerance. To this end, properties are modelled by subsets of a metric space (*X*, *d*), and a property modelled by $A \subseteq X$ is considered to imply a property $B \subseteq X$ to the degree *d* if $A \subseteq U_d(B)$, where $U_d(\cdot )$ denotes the *d*-neighbourhood.

We note that the framework proposed in Ruspini (1991) allows some additional flexibility. Instead of a metric, the central notion is a $\odot$-similarity function, where $\odot$ is a fixed t-norm (Klement et al. 2000). A $\odot$-similarity function is a mapping $s :X \times X \rightarrow [0,1]$ such that, for any $a, b, c \in X$, (i) $s(a,b) = 1$ iff $a = b$, (ii) $s(a,c) \leqslant s(a,b) \odot s(b,c)$, and (iii) $s(a,b) = s(b,a)$. Under the correspondence $$[0,1] \rightarrow \bar{\mathbb R}^+,s \mapsto {{\left\{ \begin{array}{ll} -\ln s &{} \text {if s > 0,} \\ \infty &{} \text {if s = 0}\end{array}\right. }}$$ we see that a metric can be regarded as the same as a $\odot$-similarity if $\odot$ is the product t-norm.

In order to realise Ruspini’s concept, a number of logics have been proposed in the literature (Dubois et al. 1997; Esteva et al. 1997; Rodríguez 2002; Godo and Rodríguez 2008; Esteva et al. 2012). For instance, logics endowed with a set of modal operators were studied, indexed by the elements of the real unit interval and interpreted by the neighbourhood in similarity spaces. Furthermore, a formalism using a more restricted language and getting along without modalities was introduced in Rodríguez (2002). It is based on a graded version of the implication, and it is the calculus to be considered in the present paper.

We define the *Logic of Approximate Entailment*, or $\mathsf {LAE}$ for short, as follows. The language of $\mathsf {LAE}$ includes *variables* $\varphi _0, \varphi _1, \ldots$ and the two constants $\bot$ (false), and $\top$ (true). A *Boolean formula* is built up from the variables and the constants by means of the operations $\wedge$ (and), $\vee$ (or), and $\lnot$ (not). Furthermore, a *graded implication*, or an *implication* for short, is a triple consisting of two Boolean formulas $\alpha$ and $\beta$ as well as a number $d \in {\mathbb R}^+$, denoted

$$\begin{aligned} \alpha \mathop {\rightarrow }\limits ^{d} \beta . \end{aligned}$$

A *model* for $\mathsf {LAE}$ is a metric space (*X*, *d*). An *evaluation* in a model *X* is a mapping *v* from the Boolean formulas to ${{\mathcal P}}(X)$ such that $v(\alpha \wedge \beta ) = v(\alpha ) \cap v(\beta )$, $\;v(\alpha \vee \beta ) = v(\alpha ) \cup v(\beta )$, $\;v(\lnot \alpha ) = \complement v(\alpha )$, $\;v(\bot ) = \varnothing$, and $v(\top ) = X$. We then say that *v* *satisfies* an implication $\alpha \mathop {\rightarrow }\limits ^{d} \beta$ if

$$\begin{aligned} e_d(v(\alpha ), v(\beta )) \leqslant d. \end{aligned}$$

A theory is a set of implications. We say that a theory ${\mathcal T}$ *semantically entails* an implication $\Phi$ in $\mathsf {LAE}$ if any evaluation *v* in some model *X* that satisfies all elements of ${\mathcal T}$ also satisfies $\Phi$.

We note that also modified versions of $\mathsf {LAE}$ have been considered. The present definition has been chosen in best possible accordance with the framework originally proposed in Ruspini (1991).

An axiomatisation of $\mathsf {LAE}$ is a non-trivial issue. To some extent, the mentioned modifications are a consequence of these difficulties. In Rodríguez (2002), for instance, the language is assumed to contain a fixed finite set of variables. This restriction was discarded in Vetterlein (2015); however, the assumption of symmetry of the distance function was dropped in turn.

Let us recall the calculus proposed in Vetterlein (2015). This calculus is quite similar to the axiomatic system that has been shown to be sound and complete for the finitary version of $\mathsf {LAE}$ (Rodríguez 2002; Godo and Rodríguez 2008; Esteva et al. 2012). A difference, however, is that the syntactical features relying on a fixed finite number of variables are not available here.

### Definition 4.1

$\mathbf {LAE}$ consists of the following axiom and rules, where $\alpha$, $\beta$, $\gamma$ are any Boolean formulas and $c, d \in {\mathbb R}^+$:

$$\begin{aligned}&\text {(R1)} \alpha \mathop {\rightarrow }\limits ^{0} \beta \text { if } \alpha \rightarrow \beta \hbox {is a tautology of } \mathsf {CPL}\\&\text {(R2)} \frac{\alpha \mathop {\rightarrow }\limits ^{0} \beta }{\alpha \wedge \gamma \mathop {\rightarrow }\limits ^{0} \beta \wedge \gamma }\\&\text {(R3)} \frac{\alpha \mathop {\rightarrow }\limits ^{c} \beta }{\alpha \mathop {\rightarrow }\limits ^{d} \beta }\quad \text {where }\; d \geqslant c \qquad \text {(R4)} \frac{\alpha \mathop {\rightarrow }\limits ^{c} \bot }{\alpha \mathop {\rightarrow }\limits ^{0} \bot }\\&\text {(R5)} \frac{\alpha \mathop {\rightarrow }\limits ^{c} \gamma \quad \beta \mathop {\rightarrow }\limits ^{c} \gamma }{\alpha \vee \beta \mathop {\rightarrow }\limits ^{c} \gamma } \qquad \text {(R6)} \frac{\alpha \mathop {\rightarrow }\limits ^{c} \beta \quad \beta \mathop {\rightarrow }\limits ^{d} \gamma }{\alpha \mathop {\rightarrow }\limits ^{c + d} \gamma } \end{aligned}$$

Let ${\mathcal T}$ be a theory and $\Phi$ be an implication. A *proof* of $\Phi$ from ${\mathcal T}$ in $\mathbf {LAE}$ is defined in the expected way.

We have shown in Vetterlein (2015) that $\mathbf {LAE}$ is sound and complete for an entailment relation that is closely related to $\mathsf {LAE}$. Namely, we have considered in Vetterlein (2015) the logic $\mathsf {LAE}^q$. This logic is defined similarly to $\mathsf {LAE}$. The syntax of $\mathsf {LAE}^q$ is actually defined in the same way as for $\mathsf {LAE}$. The semantic entailment relation, however, is modified as follows. A model for $\mathsf {LAE}^q$ is any *quasi*metric space (*X*, *q*), and an evaluation *v* satisfies an implication $\alpha \mathop {\rightarrow }\limits ^{d} \beta$ if $e_q(v(\alpha ), v(\beta )) \leqslant d$.

### Theorem 4.2

Let ${\mathcal T}$ be a finite theory and $\Phi$ an implication. Then ${\mathcal T}$ proves $\Phi$ in $\mathbf {LAE}$ if and only if ${\mathcal T}$ semantically entails $\Phi$ in $\mathsf {LAE}^q$.

We make now use of the results of Sect. 3 to show that $\mathbf {LAE}$ is in fact also complete with respect to metric spaces.

### Theorem 4.3

Let ${\mathcal T}$ be a finite theory and $\Phi$ an implication. Then ${\mathcal T}$ proves $\Phi$ in $\mathbf {LAE}$ if and only if ${\mathcal T}$ semantically entails $\Phi$ in $\mathsf {LAE}$.

### Proof

The soundness part holds by Theorem 4.2. To see completeness, assume that ${\mathcal T}$ does not prove $\Phi$ in $\mathbf {LAE}$. By Theorem 4.2, there exists a quasimetric space (*X*, *q*) and an evaluation *e* in (*X*, *q*) such that *e* satisfies all elements of ${\mathcal T}$ but not $\Phi$.

Let $\iota :X \rightarrow {{\mathcal P}}(Y)$ be the representation of (*X*, *q*) by means of a metric space (*Y*, *d*) according to Theorem 3.6. Then the natural extension of $\iota$ to ${{\mathcal P}}(X)$ preserves the set-theoretic operations as well as the Hausdorff quasidistance. The assertion follows. $\square$

We conclude this section with some additional notes. We first remark that the metric spaces used in the present context are of a particular nature. This observation can be seen as a consequence of the fact that Theorem 4.3 is restricted to finite theories.

### Remark 4.4

We have shown in Vetterlein (2015) the following stronger version of the completeness Theorem 4.2: assume that the finite theory ${\mathcal T}$ does not prove the implication $\Phi$ in $\mathbf {LAE}$. Then there exists an evaluation in a quasimetric space (*X*, *q*) that satisfies all elements of ${\mathcal T}$ but not $\Phi$. Moreover, the range of $q :X \times X \rightarrow \bar{\mathbb R}^+$ contains a smallest nonzero element.

As a consequence of the last-mentioned fact, the topology on (*X*, *q*) is discrete. By Lemma 3.4, so is the topology of the metric space (*Y*, *d*) representing (*X*, *q*). In other words, Theorem 4.3 remains valid when restricting the models to metric spaces whose induced topology is discrete.

We finally raise the question how our completeness theorem is related to those presented in previous works.

### Remark 4.5

As mentioned above, several completeness results have been established for a finitary version of $\mathsf {LAE}$ (Rodríguez 2002; Godo and Rodríguez 2008; Esteva et al. 2012). In that case, the language is assumed to contain a fixed finite set of variables and certain axioms rely on maximal elementary conjunctions, or m.e.c.s for short. A m.e.c. is a conjunction of literals such that all variables occur exactly once.

We may say that $\mathbf {LAE}$ arises from the axiomatic system proposed, for example, in Esteva et al. (2012) essentially by dropping the axioms containing m.e.c.s. Hence, it is natural to ask whether, in some sense, the latter are provable in $\mathbf {LAE}$. This is, however, not the case.

For instance, the axiom (A5) from Esteva et al. (2012, Def. 4.2) expresses the symmetry of the distance between the subsets interpreting two m.e.c.s. The axiom is sound because the m.e.c.s are assumed to be interpreted by sets containing at most one element. In the present context, in contrast, the conjunction of finitely many literals can be interpreted by a set of an arbitrary size; hence, symmetry cannot be expected here.

It follows in particular that the completeness theorems obtained in the mentioned previous works are not special cases of the results presented here.

## Conclusion

Quasimetrics have often be considered from the topological angle: under which conditions is a topology quasimetrisable? Assuming the more general version of the separation axiom, P. Vitolo has shown that this is the case if and only if the space is embeddable into a hyperspace of closed sets endowed with the Hausdorff quasidistance (Vitolo 1995). Quasimetrics moreover play a role for logical calculi generalising classical propositional logic, for instance in fuzzy logic (Gerla 2004) or in approximate reasoning (Ruspini 1991; Rodríguez 2002). The present paper is devoted to the last-mentioned aspect.

We have presented a representation theorem for quasimetric spaces that is, in a sense, analogous to Vitolo’s. However, we have dealt with quasimetric spaces in a narrower sense and could then enhance the representation by additional features. In this way, an open issue concerning the Logic of Approximate Entailment could be solved. For this logic, defined closely to its original formulation by Ruspini, a sound and complete axiomatisation had not been known. Based on our result on quasimetric spaces, we were able to fill this gap.

A further progress in the field of approximate reasoning remains certainly desirable in several respects. For instance, our completeness theorem is for finite theories only; the question is open if the finiteness condition could be dropped. To this end, the complexity of the procedure of proving completeness, contained in our previous paper (Vetterlein 2015) as well as in the present one, might be worthy of consideration. A non-trivial amount of partly technically demanding auxiliary facts were required, and it is open if there is an alternative, more direct way. Furthermore, we have considered by now Rodríguez’s Logic of Approximate Entailment only. It might be interesting to apply the same methods to related logics. Fuzzy logics or logics aiming at the formalisation of vague statements might well be considered in a framework similar to the one considered in this paper and might offer new perspectives in both formal and informal respects.
